# Supplementary material for: A critical role of a eubiotic microbiota in gating proper immunocompetence in Arabidopsis
Source: Nat Plants. 2023 Aug 17;9(9):1468–80. doi: 10.1038/s41477-023-01501-1 (PMC10505558; doi:10.1038/s41477-023-01501-1)
Supplement: Supplementary file 1 — Reporting Summary [file 41477_2023_1501_MOESM1_ESM.pdf]

nature portfolio

Corresponding author(s): Sheng Yang He

Last updated by author(s): Jul 21, 2023

Reporting Summary

Nature Portfolio wishes to improve the reproducibility of the work that we publish. This form provides structure for consistency and transparency in reporting. For further information on Nature Portfolio policies, see our Editorial Policies and the Editorial Policy Checklist.

Statistics

For all statistical analyses, confirm that the following items are present in the figure legend, table legend, main text, or Methods section.

n/a	Confirmed	
☐	☒	The exact sample size (*n*) for each experimental group/condition, given as a discrete number and unit of measurement
☐	☒	A statement on whether measurements were taken from distinct samples or whether the same sample was measured repeatedly
☐	☒	The statistical test(s) used AND whether they are one- or two-sided<br>*Only common tests should be described solely by name; describe more complex techniques in the Methods section.*
☒	☐	A description of all covariates tested
☐	☒	A description of any assumptions or corrections, such as tests of normality and adjustment for multiple comparisons
☐	☒	A full description of the statistical parameters including central tendency (e.g. means) or other basic estimates (e.g. regression coefficient) AND variation (e.g. standard deviation) or associated estimates of uncertainty (e.g. confidence intervals)
☐	☒	For null hypothesis testing, the test statistic (e.g. *F*, *t*, *r*) with confidence intervals, effect sizes, degrees of freedom and *P* value noted<br>*Give P values as exact values whenever suitable.*
☒	☐	For Bayesian analysis, information on the choice of priors and Markov chain Monte Carlo settings
☒	☐	For hierarchical and complex designs, identification of the appropriate level for tests and full reporting of outcomes
☒	☐	Estimates of effect sizes (e.g. Cohen's *d*, Pearson's *r*), indicating how they were calculated

Our web collection on statistics for biologists contains articles on many of the points above.

Software and code

Policy information about availability of computer code

Data collection	Gene expression: ABI 7500 SDS v2.0, QuantStudio Design and Analysis v1.5.2<br>RNA-seq: Illumina Real Time Analysis (RTA) version 1.18.64, Illumina RTA 3<br>ROS: Molecular Devices SoftMax Pro v7.0.3<br>Western blots: Invitrogen iBright 1500 system<br>Hormone quantification: Waters MassLynx v4.1, Waters MassLynx v4.1 QuanOptimize<br>16S: Illumina RTA v1.18.54, Illumina Bcl2fastq v2.20.0
Data analysis	Statistics and graph production: GraphPad Prism 10.0.0 software<br>Gene expression: QuantStudio Design and Analysis v1.5.2<br>RNA-seq: Illumina Bcl2fastq v2.20.0, FastQC, Trimmomatic, STAR v9.3.0, Rsubreads version 2.8.2, DESeq2, EdgeR on the iDEP platform v1.0, ShinyGO version 0.76.2, https://github.com/rsohrabi/MIP_ms<br>Total ROS: GraphPad Prism software version 10.0.0<br>Protein quantification: ImageJ v1.51<br>Hormone quantification: Waters MassLynx v4.1, Waters MassLynx v4.1 QuanOptimize<br>16S amplicon analysis: QIIME 2 Core 2022.2 distribution (including DADA2 and RESCRIPt plugins), Cutadapt v4.1, https://github.com/BradCP/A-critical-role-of-a-eubiotic-microbiota-in-gating-proper-immunocompetence-in-Arabidopsis

For manuscripts utilizing custom algorithms or software that are central to the research but not yet described in published literature, software must be made available to editors and reviewers. We strongly encourage code deposition in a community repository (e.g. GitHub). See the Nature Portfolio guidelines for submitting code & software for further information.

Data

Policy information about availability of data

All manuscripts must include a data availability statement. This statement should provide the following information, where applicable:

- Accession codes, unique identifiers, or web links for publicly available datasets
- A description of any restrictions on data availability
- For clinical datasets or third party data, please ensure that the statement adheres to our policy

The RNA-seq raw sequencing and analyzed data have been deposited in the NCBI Gene Expression Omnibus database under accession GSE218961 and GSE218962. Raw source 16S rRNA gene sequences from this project are available in the Sequence Read Archive database under BioProject PRJNA977816, accession numbers SAMN35534885 to SAMN35534914. QIIME-compatible SILVA 16S rRNA gene reference sequences and taxonomy (release 138) can be downloaded from https://docs.qiime2.org/2022.2/data-resources/. Source data are provided with this paper.

Human research participants

Policy information about studies involving human research participants and Sex and Gender in Research.

Reporting on sex and gender	N/A
Population characteristics	N/A
Recruitment	N/A
Ethics oversight	N/A

Note that full information on the approval of the study protocol must also be provided in the manuscript.

Field-specific reporting

Please select the one below that is the best fit for your research. If you are not sure, read the appropriate sections before making your selection.

☒ Life sciences ☐ Behavioural & social sciences ☐ Ecological, evolutionary & environmental sciences

For a reference copy of the document with all sections, see nature.com/documents/nr-reporting-summary-flat.pdf

Life sciences study design

All studies must disclose on these points even when the disclosure is negative.

Sample size	Sample size and statistical analyses are described in the relevant figure legends. Sample size was determined based on experimental trials and with consideration of previous publications (Chen et al. 2020 PMID: 32350464) on similar experiments to allow for confident statistical analyses. There were no statistical methods used to predetermine sample sizes.
Data exclusions	No data that pass quality control were excluded from statistical analysis.
Replication	The number of independent replication for each experiment is described in the relevant figure legends. Two or more independent experiments were performed for all assays. Results were ensured to be reproducible in all repeats with the same trend.
Randomization	Gnotobiotic plants were grown within Microboxes placed side-by-side in environmentally-controlled growth chambers (light, temperature, humidity) to control other covariates and to minimized unexpected environmental variations. Additionally, Microboxes were rotated periodically to randomize the effect of localized environmental differences within a chamber. Leaf samples of similar age were collected from plants at the indicated ages and assessed randomly.
Blinding	Researchers were not blinded to allocation during experiments and outcome assessment. This is in part because gnotobiotic plants with different treatments can exhibit phenotypes than makes them identifiable visually. Thus, blinding was not possible in these cases. Routine practices included more than one author observing/assessing phenotypes, whenever possible.

Reporting for specific materials, systems and methods

We require information from authors about some types of materials, experimental systems and methods used in many studies. Here, indicate whether each material, system or method listed is relevant to your study. If you are not sure if a list item applies to your research, read the appropriate section before selecting a response.

Materials & experimental systems

n/a	Involved in the study
☐	☒ Antibodies
☒	☐ Eukaryotic cell lines
☒	☐ Palaeontology and archaeology
☒	☐ Animals and other organisms
☒	☐ Clinical data
☒	☐ Dual use research of concern

Methods

n/a	Involved in the study
☒	☐ ChIP-seq
☒	☐ Flow cytometry
☒	☐ MRI-based neuroimaging

Antibodies

Antibodies used

The antibodies used in this study are commercially available: anti-BAK1 (Agrisera AS12 1858, used at 1:5000), anti-FLS2 (Agrisera AS12 1857, used at 1:5000), anti-MPK3 (Sigma M8318, used at 1:500), anti-MPK6 (Sigma A7104, used at 1:2000), anti-phospho-p44/42 MAPK Erk1/2 Thr202/Tyr204 (Cell Signaling 9101, used at 1:1000), Goat anti-rabbit HRP (Agrisera AS09 602, used at 1:40000).

Validation

All antibodies used in this study are commercially available and validated according to the manufacturers specifications:
anti-BAK1 - https://www.agrisera.com/en/artiklar/bak1-bri1-associated-receptor-kinase.html
anti-FLS2 - https://www.agrisera.com/en/artiklar/fls2-flagellin-sensitive-2-.html
anti-MPK3 - https://www.sigmaaldrich.com/US/en/product/sigma/m8318
anti-MPK6 - https://www.sigmaaldrich.com/US/en/product/sigma/a7104
anti-phospho-p44/42 MAPK - https://www.cellsignal.com/products/primary-antibodies/phospho-p44-42-mapk-erk1-2-thr202-tyr204-antibody/9101
anti-rabbit - https://www.agrisera.com/en/artiklar/goat-anti-rabbit-igg-hl.html
