# Supplementary figures and images for: A critical role of a eubiotic microbiota in gating proper immunocompetence in Arabidopsis
Source: Nat Plants. 2023 Aug 17;9(9):1468–80. doi: 10.1038/s41477-023-01501-1 (PMC10505558; doi:10.1038/s41477-023-01501-1)

Source data for Fig. 3c

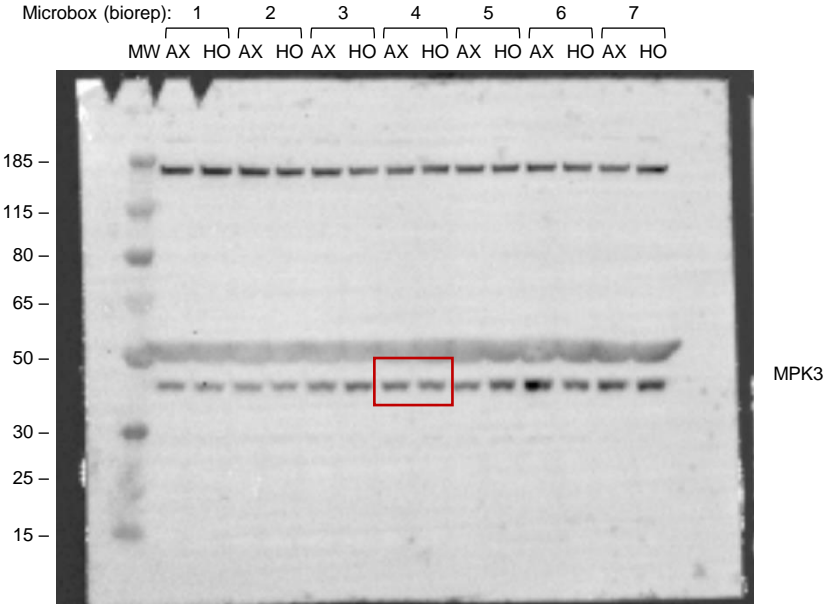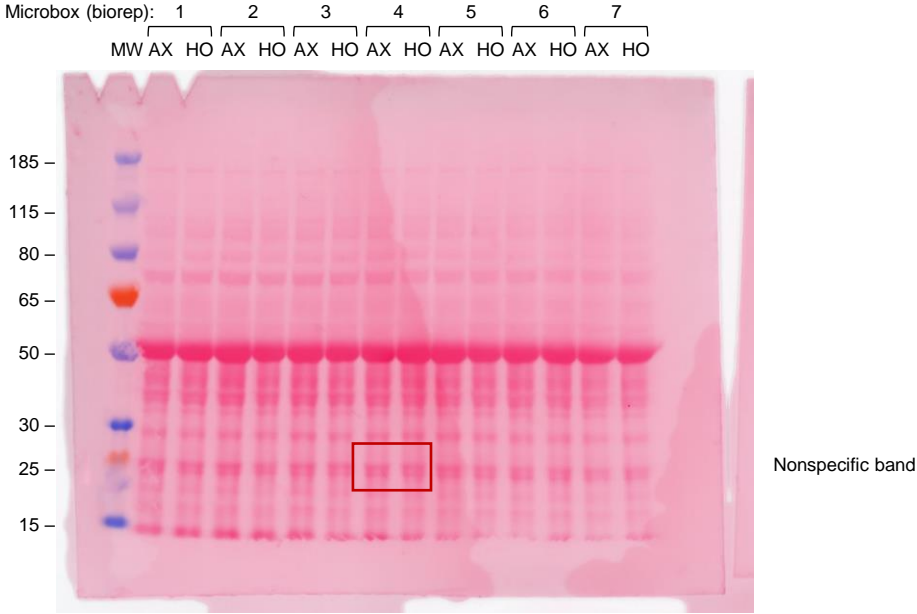

Source data for Fig. 3d

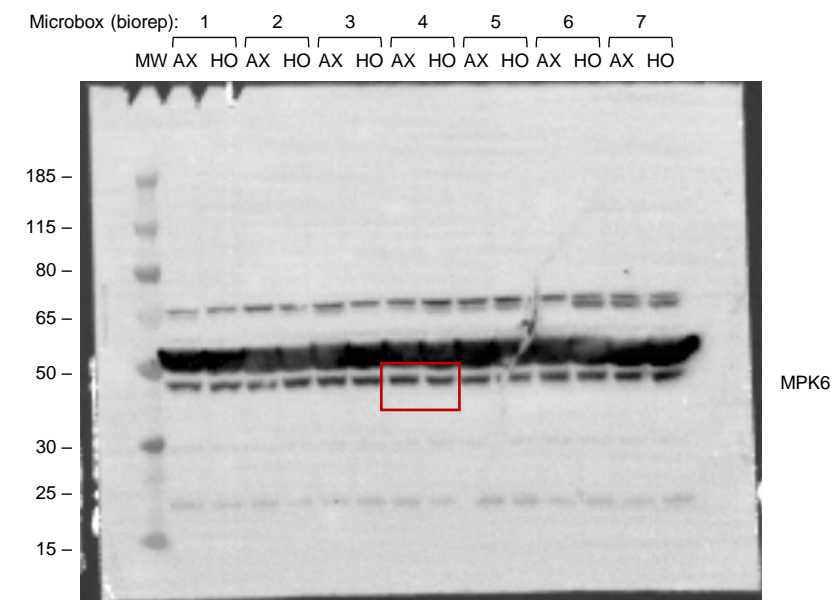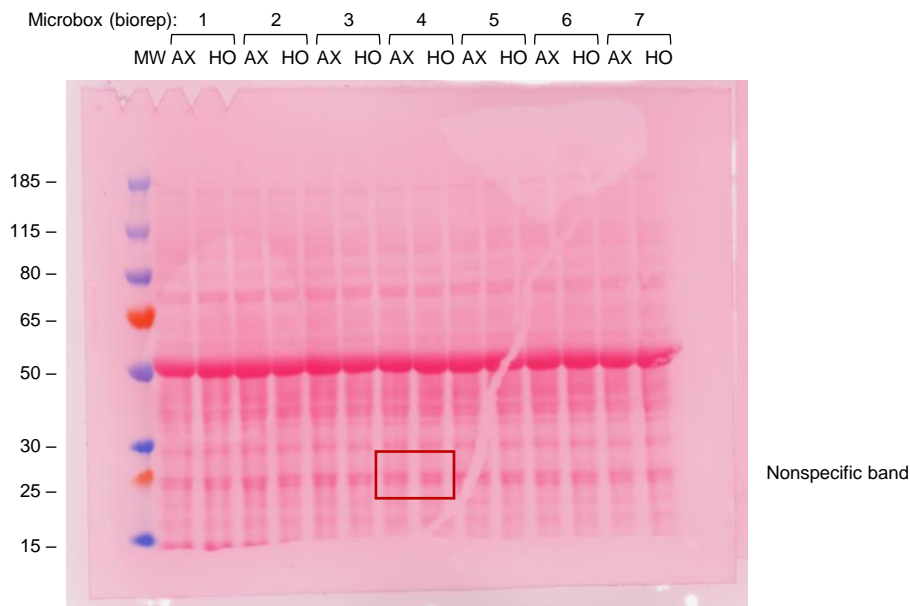

Source data for Fig. 3e

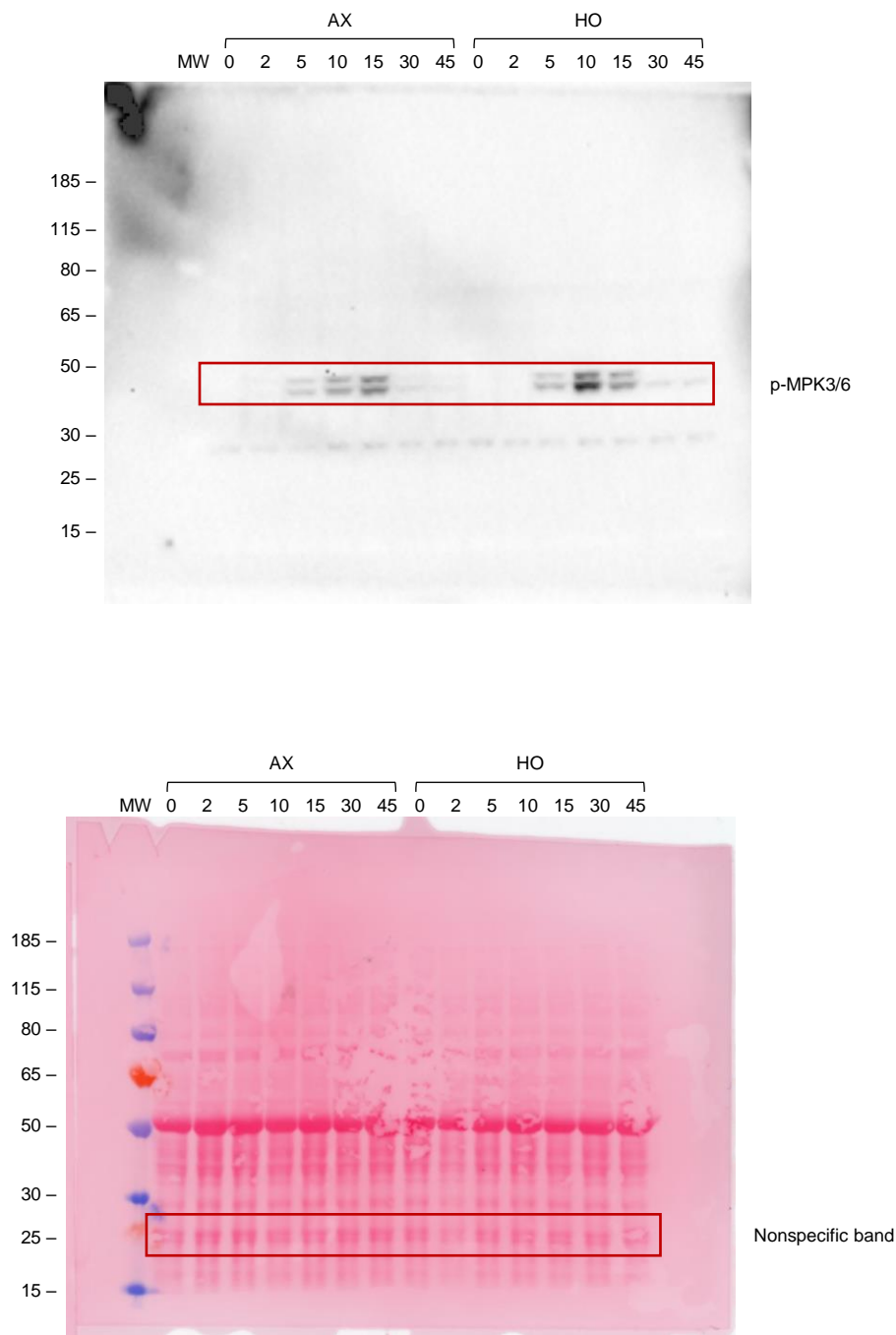

Source data for Fig. 3g

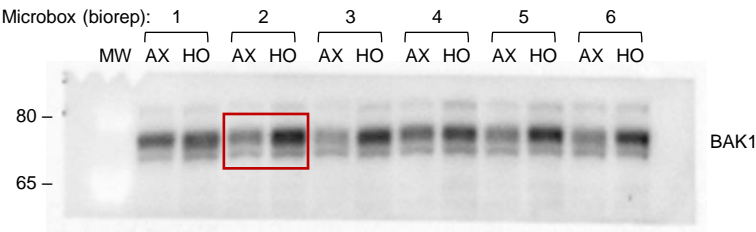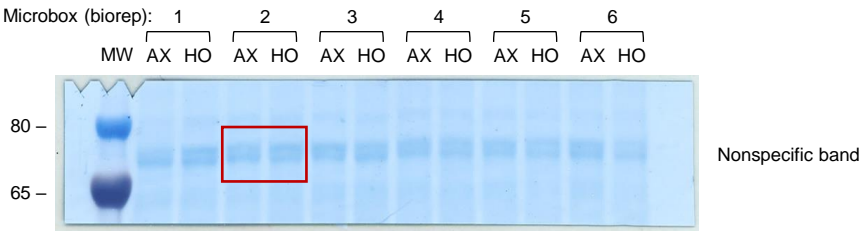

Supplement: Source Data Fig. 3 — Unprocessed western blots. [file 41477_2023_1501_MOESM5_ESM.pdf]

Source data for Extended Data Fig. 3

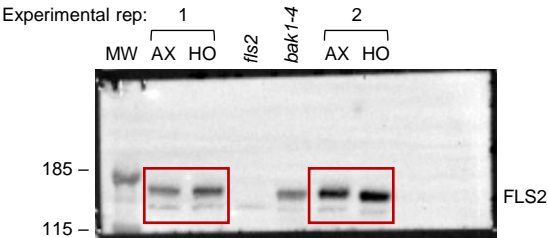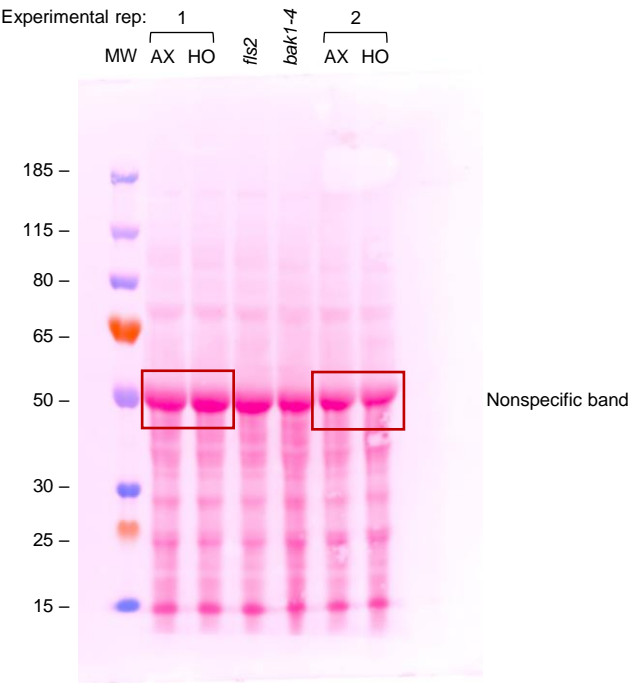

Supplement: Source Data Extended Data Fig. 3 — Unprocessed western blots. [file 41477_2023_1501_MOESM12_ESM.pdf]

Source data for Extended Data Fig. 5b

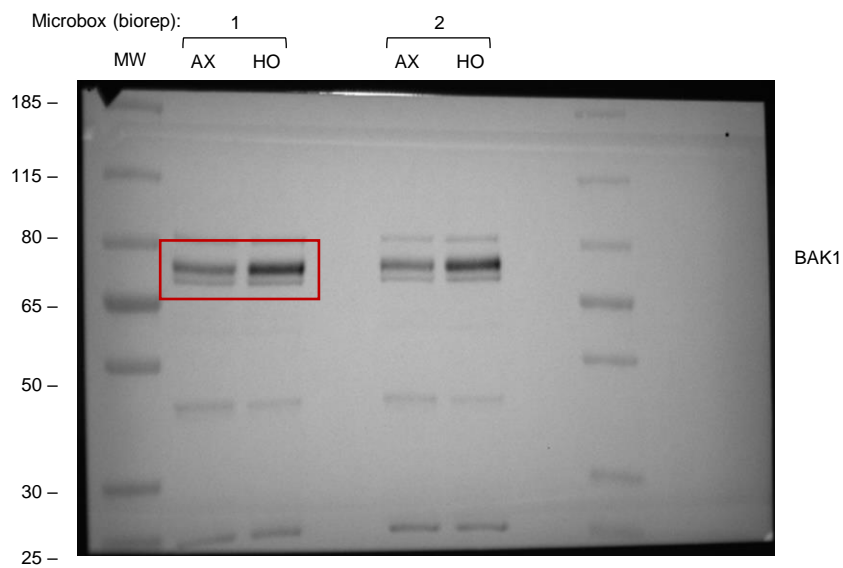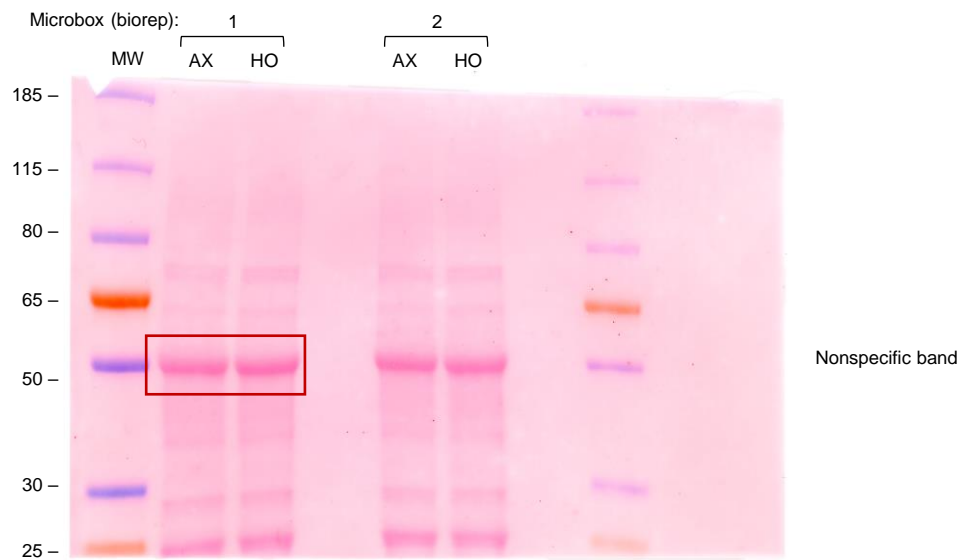

Supplement: Source Data Extended Data Fig. 5 — Unprocessed western blots. [file 41477_2023_1501_MOESM15_ESM.pdf]
